# Examination of Failures in Vaccination

**Published:** 1804-06-01

**Authors:** John Walker

**Affiliations:** Salisbury Square


### *To the Editors of the Medical and Physical Journal.*

GENTLEMEN,

THE pamphlet of "William Goldson, Member of the Royal College of Surgeons in London," having this evening fallen into my hands, I offer you some Remarks on it; it; to which, if you think them worthy of insertion, I may hereafter make some additions.

The first thing I am struck with is the boldness of the author. His title is, "Cases of Small-pox subsequent to Vaccination." There is at this time a Committee appointed by the Royal Jennerian Society (including a Woodville and a Ring, than which names it would be difficult to find greater authorities in the history of Variola and Vacciola) to investigate *a case* which the author would doubtless not hesitate to rank with those of his pamphlet. Being on this Committee, I have had the opportunity of observing with what becoming modesty and caution men of the greatest experience and character offer their opinions, and I cannot but think that the title of the pamphlet under my eye, would have been only correct if it had been expressed, after *supposed* cases of Vaccination. The practitioner who inoculated in *the case* yet *sub investigatione*, informed me that as he had not taken minutes of it at the time, and did not now remember it, he supposed it had been a spurious case; and I persuade myself that *the different cases* of W. G. must all have been spurious cases. But, let me turn to the pamphlet.

Page 9. "If we (they of Portsmouth, Portsea, and Gosport) were not so early in adopting it (Vaccination) as in places less remote from London, we have embraced one very material advantage by our forbearance—we have experienced a much smaller number of failures. In no instance, have I myself seen any approach to a spurious disease; and very few indeed, to my knowledge, have occurred, &c."

"But the occurrence of small-pox in Clark, one of the marines first of all inoculated at the Infirmary by Mr. Rickman, seemed to give some check to the practice, &c."

Page 8. "With matter taken from this source (the Infirmary) I inoculated four children. And in a very short space of time it was pretty generally adopted in the neighbourhood." How could any but spurious cases be expected from the source which failed to yield protection to the marine?

Page 16. The author says of one case, "Several medical gentlemen saw the child with me, who were decidedly of opinion as to the nature of the eruptions.—That they were variolous." When a man goes to oppose his own to a general opinion, he ought to be very clear in the establishment of every statement. Why does not he give the names of his medical gentlemen? It ought not to to have been a fortnight or more after his inoculation with variolous matter before the medical gentlemen visited the child. From his own description of the case, they must have possessed very superior powers of discrimination to have been able to distinguish the nature of the remaining eruptions, covered with a warty scurf or encrustation, four days before their seeing them. I think his case of small-pox must have been altogether spurious. But, *passons* to the cases of the author's own vaccination.

Page 25. "The pustule was perfectly satisfactory, &c." To whom? Here we have not even the concurrence of anonymous medical men to substantiate the author's conclusion. On this subject, above three years afterwards, "seven distinct eruptions appeared on the face, neck, and arms. These were so characteristic of the small-pox, that neither Mr. Hill, Mr. Seeds, or Mr. Weymouth, three respectable practitioners in Portsea, who saw the child with me, hesitated to pronounce them the effect of variolous contagion."

Had these gentlemen seen the child under its supposed vaccination, they would probably have had to declare, *it must be re-inoculated, it is not yet protected.* In this way I have myself had to damp the joy of surgeons up the Mediterranean both Spanish and Italian. They have shewn me finely inflamed looking arms; but I had been already too much harrassed with spurious cases to hesitate for a moment in pronouncing the effects altogether spurious.

Page 29. "The progress of the arm was extremely regular, &c." "Matter was taken from the pustule early on the ninth day, which I used on a child who had the disease nearly in the same manner, &c." "Mr. Merritt, of Portsmouth likewise vaccinated a child with matter from the same source." Did he see the pock from which it was taken? If so, he must have had an eye as little experienced in detecting spurious pustule as W. G. or the surgeons up the Mediterranean. The author has clearly shewn that the child had the small-pox three years afterwards.

These are not all the cases of small-pox which have occurred after supposed cases of Vaccination, *in the place where they have less than in others experienced failures;* but I have not at present leisure to go further into the subject.

*Salisbury Square,* 28, *iv.* 1804. JOHN WALKER.

P.S. 5, v. 1804. An application has just been made at the Central House of the Royal Jennerian Society for vaccine matter from "Dr. Waller, of Portsmouth," with the remark, that that which they have there cannot be depended pended on; and on that account he sends to the Society, where he reckons upon being furnished with the genuine. Thus we find that, happily, all the medical men in that neighbourhood have not, in suspicious cases, the full confidence of W. G. Let us hope then that spurious pustule will there disappear, and that the inhabitants will hereafter have only to do with the certain protection.

11, v. 1804. I have just been called upon for another packet of vaccine matter for "Mr. Shute, of Gosport," and have sent a rich supply, which may produce more *heartfelt* conviction in those parts than long arguments.

